# Mechanistic insights into cisplatin-induced ototoxicity: the central role of transient receptor potential channels

**DOI:** 10.3389/fmolb.2025.1694131

**Published:** 2025-10-15

**Authors:** Man Yu, Juanjuan Li, Xianhai Zeng

**Affiliations:** ^1^ Department of Graduate and Scientific Research, Zunyi Medical University Zhuhai Campus, Zhuhai, Guangdong, China; ^2^ Department of Otolaryngology, Longgang Otolaryngology Hospital and Shenzhen Institute of Otolaryngology, Shenzhen, Guangdong, China

**Keywords:** transient receptor potential channels, cisplatin, cochlear hair cells, calcium, therapy

## Abstract

This review systematically elucidates the molecular mechanisms underlying cisplatin-induced ototoxicity, with a particular focus on the pivotal role played by TRP channels. First, the article outlines the uptake and efflux pathways of cisplatin within cochlear hair cells, followed by a detailed analysis of the core mechanisms by which cisplatin damages these cells. It emphasizes the structural and functional characteristics of TRP channels and their action mechanisms in cisplatin ototoxicity, elucidating the channels' high selectivity for calcium ions and their central role in driving ototoxicity. Additionally, the review explores the roles of other TRP family members in regulating hair cells. Finally, based on an analysis of the limitations of existing otoprotective strategies, the review proposes future precision prevention and treatment strategies targeting TRP channels. These include novel nanodelivery technologies and multi-pathway combined interventions, providing a new theoretical foundation and translational direction for protecting against cisplatin ototoxicity.

## 1 Introduction

Ototoxicity is the irreversible destruction of inner-ear structures-hair cells, spiral ganglion neurons (SGNs), and supporting tissues-elicited by drugs or chemicals, culminating in sensorineural hearing loss (SNHL) or vestibular impairment (Kros and Steyger, 2019). The World Health Organization’s 2021 report underscores the global burden: 1.5 billion people-one in five-live with hearing loss, 430 million of whom have disabling impairment (>40 dB). Unmitigated hearing loss siphons an estimated US $980 billion annually from the world economy, and projections indicate that by 2050 more than 700 million individuals will suffer disabling deficits (Chadha et al., 2021). Among preventable etiologies, pharmacologic ototoxicity looms large. The cochlea uniquely retains platinum-based drugs for months to years after systemic exposure in both murine models and humans (Breglio et al., 2017). Consequently, over 500,000 patients develop chemotherapy-related hearing impairment each year (Dillard et al., 2022). Children are especially susceptible: 30%–60% of pediatric cancer survivors treated with cisplatin sustain permanent high-frequency SNHL (Brock et al., 2018). Ototoxic injury is both frequency-selective-cisplatin preferentially annihilates basal, high-frequency OHCs-and cumulative across treatment cycles (Rybak et al., 2019). Transient receptor potential (TRP) channels are abundantly expressed throughout the cochlea, where they govern auditory transduction, mechanosensation, and synaptic transmission. Best known for mediating taste, touch, and olfaction across phylogeny, these polymodal sensors are activated by an array of environmental and endogenous ligands (Damann et al., 2008). TRP channels conduct Ca^2+^, Na^+^, K^+^, and Mg^2+^, positioning them as pivotal integrators of intracellular signaling cascades (Tsagareli and Nozadze, 2020). Emerging evidence indicates that TRP channels also constitute critical entry portals and signal amplifiers for cisplatin-induced cochlear injury. This review focuses on the structural and functional characteristics of TRP channels, detailing the mechanism by which cisplatin enters hair cells in the cochlea *via* TRP channels, leading to ototoxicity. By weaving together molecular, cellular, and pre-clinical data, we delineate the pathogenic axis linking TRP channel activation to cisplatin ototoxicity and outline rational therapeutic strategies aimed at preventing or reversing cisplatin-induced hearing loss.

## 2 Pathways and mechanisms of cisplatin-induced cochlear hair cell damage

### 2.1 Uptake and efflux pathways of cisplatin in cochlear hair cells

The initial phase of cisplatin ototoxicity begins with the drug’s penetration into the inner ear and its progressive accumulation within vulnerable cochlear cells. Following systemic administration, cisplatin traverses the blood–labyrinth barrier (BLB) and gains access to the endolymph primarily *via* the stria vascularis (SV) (Wang and Steyger, 2009). The marginal cells lining the SV-key constituents of the BLB-represent early pathogenic targets: cisplatin-induced dysfunction compromises barrier integrity, increases BLB permeability, and sustains an inward flux of the drug while simultaneously igniting localized inflammatory cascades (Laurell et al., 2007; Thomas et al., 2006). Critically, cisplatin can persist within the SV for months to years, a prolonged retention that is increasingly recognized as a fundamental driver of delayed and progressive ototoxic injury (Breglio et al., 2017).

Once cisplatin breaches the BLB, it is rapidly sequestered by cochlear hair cells through multiple, partially redundant uptake routes. Beyond fluid-phase endocytosis, the principal conduits are (i) the mechano-electrical transduction (MET) channels that gate cation influx at the stereociliary tips, (ii) the organic cation transporter 2 (OCT2, SLC22A2), and (iii) the high-affinity copper transporter 1 (CTR1, SLC31A1) (Hibino et al., 2010; Sprowl et al., 2014). Immunohistochemical and transcriptomic profiling reveal robust OCT2 and CTR1 expression in outer and inner hair cells (IHCs), SGNs, and strial marginal cells (Ravi et al., 1995; Tang et al., 2021; More et al., 2010), precisely co-localizing with cisplatin-induced lesions.

Genetic or pharmacologic suppression of OCT2 markedly attenuates cochlear uptake of cisplatin and protects auditory function in pre-clinical models without compromising tumoricidal activity (Sprowl et al., 2014; Ciarimboli et al., 2010; Sprowl et al., 2013), underscoring OCT2 as a tractable otoprotective target. CTR1, however, is also abundant in many malignancies (Ishida et al., 2010), so systemic CTR1 inhibition risks diminishing chemotherapeutic efficacy. MET channels serve as the core molecular components through which hair cells perceive mechanical stimuli, acting as pathways for cisplatin and Ca^2+^ entry into hair cells. The otoprotective potential of targeting MET channels has been extensively validated through research. MET channel blockers such as benzamil and small-molecule compounds like ORC-13661 and UoS-7692 have demonstrated protective effects against cisplatin ototoxicity. However, most MET blockers remain in animal testing phases, with clinical translation still facing challenges requiring further validation of their safety and efficacy (Kitcher et al., 2019; Kenyon et al., 2021; Maruyama et al., 2024). Other calcium-permeable channels and transporters also play critical roles in cochlear physiology and pathology. The L-type voltage-gated calcium channel Cav1.3 is the most important calcium channel in IHCs, mediating calcium influx that triggers neurotransmitter release and sound signal transmission. CACNA1D knockout mice (the gene encoding Cav1.3) exhibit congenital deafness (Platzer et al., 2000). Cav3.2 is the most prominently expressed T-type voltage-gated calcium channel entity in the cochlea and auditory brainstem, holding significant functional importance for spatiotemporal auditory processing across different regions of the auditory system (Lundt et al., 2019). ATP-gated P2X (2) receptors are highly expressed in cochlear supporting cells and the SV, being essential for lifelong normal hearing and protection against noise exposure (Yan et al., 2013). The sodium-calcium exchanger (NCX), as a key transporter regulating intracellular Ca^2+^/Na^+^ concentrations, is crucial for maintaining endolymphatic ion homeostasis and inner ear potential. Its dysfunction may lead to hearing loss. Store-Operated Ca^2+^ Channels (SOCCs) are pivotal for calcium wave formation in interdenticular cells connecting inner supporting cells to SGNs. The direct association with hearing opens broad prospects for studying cochlear calcium signaling and auditory function (Ma et al., 2023). In cisplatin- and noise-induced ototoxicity models, systemic and intratympanic administration of calcium channel blockers can prevent hearing loss (Naples, 2017). Finally, cisplatin can traverse lipid bilayers *via* passive diffusion. Within the cytosol it undergoes aquation to a positively charged, membrane-impermeant species that becomes trapped intracellularly, fostering prolonged drug residence and sustained cytotoxic signaling (van Ruijven et al., 2004; Hazlitt et al., 2018; Yu et al., 2020).

Compared with the well-characterized uptake pathways, cisplatin efflux from cochlear cells remains poorly understood. Functionally, CTR2 operates chiefly as an efflux transporter: overexpression confers cellular resistance by extruding cisplatin, whereas CTR2 knockdown augments intracellular platinum accumulation and cytotoxicity (Blair et al., 2009; Blair et al., 2011). Thus, CTR2 modulation could potentiate antitumor activity while simultaneously heightening ototoxicity-a therapeutic paradox that requires nuanced intervention. Current evidence suggests that only a limited set of export pumps may modulate intracellular platinum levels. The copper-transporting P-type ATPases ATP7A and ATP7B are both expressed in the cochlea-ATP7A localizes predominantly to pillar cells of the organ of Corti, whereas ATP7B is found in hair cells-and have been implicated in tumor resistance to platinum drugs (Ding et al., 2011). Whether they actively extrude cisplatin from cochlear cells, however, has not been demonstrated. Multidrug resistance-associated proteins (ABCC2/3) and multidrug and toxin extrusion transporters (MATE1/2-K) have also been shown to mediate cisplatin efflux in hepatic and renal epithelia, influencing both systemic clearance and tissue toxicity (Nakamura et al., 2010; Wen et al., 2014). Polymorphisms such as ABCC2 rs11597282 and ABCC3 rs1051640 have been proposed to underlie inter-individual variability in platinum-associated adverse effects (Sun et al., 2010; Pussegoda et al., 2013), yet no study has directly linked these variants-or any efflux pump-to protection against cisplatin-induced ototoxicity.

### 2.2 Mechanisms of cisplatin damage to cochlear hair cells

Once inside cochlear cells, cisplatin unleashes a cascade of interconnected toxic pathways that converge on cell death. The inciting event is an overwhelming oxidative burst: cisplatin cripples the endogenous antioxidant network and selectively hyper-activates the NADPH oxidase-3 (NOX3), driving a surge of reactive oxygen species (ROS) (Shen et al., 2023). These ROS operate as primary executioners-directly oxidizing lipids, proteins, and nucleic acids-and as amplifiers that target the mitochondrial compartment, precipitating membrane depolarization and cytochrome-c release (Bonawitz et al., 2006). In parallel, ROS act as signaling molecules that ignite inflammation: they activate STAT1 and NF-κB, which transcriptionally upregulate tumor necrosis factor-α (TNF-α) and a suite of additional pro-inflammatory cytokines (Ramkumar et al., 2022). The resulting feed-forward loop, in which oxidative stress fuels inflammation and inflammatory mediators further potentiate ROS generation, seals the fate of sensory hair cells.

This self-perpetuating cycle ultimately cripples mitochondria-the linchpins of energy metabolism and cellular survival. Cochlear hair cells, with their prodigious ATP demands, are exquisitely vulnerable to mitochondrial insult. Cisplatin triggers mitochondrial DNA (mtDNA) mutations, deranges Ca^2+^ homeostasis, and provokes a secondary wave of mitochondrial ROS (mtROS) that further destabilizes the organelle (He et al., 2025; Tan and Song, 2023). Concomitantly, the drug tilts the balance of Bcl-2-family proteins: pro-apoptotic Bax is upregulated, anti-apoptotic Bcl-xL is suppressed, and the mitochondrial membrane potential collapses, releasing cytochrome c into the cytosol (Shen et al., 2023). Cytochrome c nucleates the apoptosome, activating caspase-9 and propagating a cascade that culminates in caspase-3–driven apoptosis (Wang et al., 2004). Mitochondrial Ca^2+^ overload is a critical accelerant of this process, triggering translocation of apoptosis-inducing factor and amplifying chromatinolysis (Motyl, 1999; Kuwana and Newmeyer, 2003). Intracellular aquation converts cisplatin into highly electrophilic hydrated complexes that alkylate nuclear and mitochondrial DNA, spawning additional mtROS and perpetuating a vicious damage loop (Graterol et al., 2017). This genotoxic insult also activates the p53 network, which transcriptionally upregulates Bax and directly modulates components of the caspase cascade, thereby intensifying apoptotic execution (Benkafadar et al., 2017).

Beyond canonical apoptosis, cisplatin provokes additional, mechanistically distinct modes of cell death. Recent work reveals that the drug triggers pyroptosis in strial marginal cells *via* the NLRP3 inflammasome. This entails sequential activation of caspase-1, cleavage of gasdermin-D (GSDMD), formation of plasma-membrane pores, and massive extrusion of IL-1β and DNA fragments. Silencing NLRP3 or its upstream regulator thioredoxin-interacting protein (TXNIP) with siRNA markedly attenuates this cascade, establishing the TXNIP/NLRP3/GSDMD axis as a pivotal driver of localized, inflammatory cell death (Yu et al., 2022). Concomitantly, intrinsic cytoprotective programs are mobilized. Autophagy, the primary catabolic pathway for intracellular quality control, exerts a predominantly pro-survival influence in cisplatin-exposed hair cells; enhancing autophagic flux mitigates sensory cell loss (Liu et al., 2021; Fang and Xiao, 2014; Pang et al., 2018). This protective autophagy is governed by the master transcriptional regulator TFEB, whose nuclear translocation coordinates lysosomal biogenesis and autophagosome-lysosome fusion (Li et al., 2022; Sette et al., 2011). Gap-junction-mediated intercellular communication adds another layer of defense. Activation of cAMP signaling through gap-junctional transfer elevates PKA-dependent phosphorylation of CREB, bolstering anti-apoptotic gene expression. Disruption of this network, conversely, magnifies cisplatin-induced injury (Kim et al., 2021).

Collectively, these interlaced pathways-pyroptotic inflammation, autophagic resilience, and junctional cytoprotection-form a multifaceted network that both propagates and constrains cisplatin ototoxicity, offering diverse, mechanistically grounded targets for future therapeutic intervention.

## 3 Role and mechanisms of TRP channels in cochlear hair cell damage

### 3.1 Composition and function of TRP channels

**FIGURE 1:**
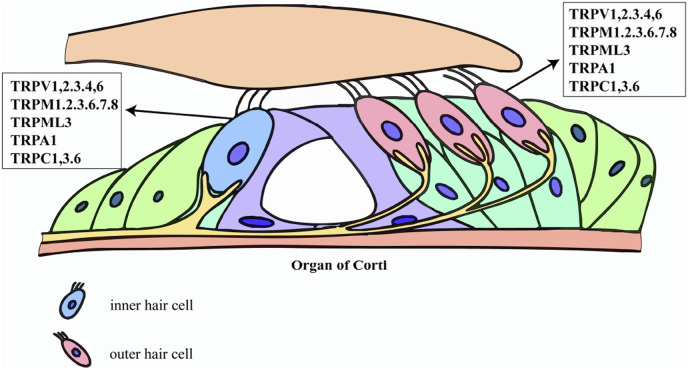
Expression map of TRP channels in cochlear hair cells. This schematic illustrates the specific distribution of key transient receptor potential (TRP) channels identified in inner hair cells (IHCs) and outer hair cells (OHCs). The figure highlights the presence of multiple TRP subfamily members-including TRPV, TRPM, TRPML, TRPA, and TRPC family members-on these cells. This selective expression supports their critical roles in hair cell physiology and involvement in mechanisms such as cisplatin-induced ototoxicity.

TRP channels constitute a ubiquitous superfamily of non-selective cation pores-most notably permeable to Ca^2+^-that are expressed across virtually every mammalian tissue. Their name derives from the original discovery in *Drosophila* visual-transduction mutants (Montell and Rubin, 1989). Architecturally, TRP channels are built on a six-transmembrane (S1–S6) blueprint; a re-entrant pore loop situated between S5 and S6 governs cation selectivity (Wu et al., 2010). Phylogenetic and functional criteria divide the superfamily into seven principal subfamilies: TRPA (ankyrin), TRPC (canonical), TRPM (melastatin), TRPML (mucolipin), TRPP (polycystin), TRPV (vanilloid), and TRPN (NOMPC). A defining hallmark is their polymodal activation. TRP channels integrate physical cues-temperature, membrane stretch, osmotic stress-with an expansive chemical lexicon that includes oxidized lipids, inflammatory mediators, and endogenous metabolites (Venkatachal et al., 2007). Thus, TRPV1 responds to noxious heat (>43 °C), capsaicin, or acidic pH, whereas TRPM8 is gated by mild cold and menthol (McKemy et al., 2002; Caterina et al., 1997). Several members, exemplified by TRPM6 and TRPM7, are “chanzymes,” bearing a C-terminal enzymatic domain that endows them with simultaneous ion-channel and kinase activities (Nadler et al., 2001). This structural and functional versatility positions TRP channels as central integrators of sensory transduction and cellular stress responses. Within the auditory system, TRP channels act as polymodal sentinels that govern mechano-electrical transduction, Ca^2+^ homeostasis, developmental patterning, and stress adaptation by integrating an array of endogenous and environmental cues (Gees et al., 2012). TRPV4 is prominently expressed in both hair cells and adjacent supporting cells, where it senses osmotic fluctuations and stabilizes the endolymphatic microenvironment (Shen et al., 2006). Members of the TRPC subfamily-most notably TRPC3 and TRPC6-reside in SGNs and cochlear hair cells, fine-tuning Ca^2+^ influx and thereby modulating mechanotransduction fidelity (Quick et al., 2012). TRPML3 localizes to lysosomes and to the basal pole of stereocilia in hair cells, coupling lysosomal trafficking and organellar quality control to auditory function (Casti et al., 2011). TRP channel activity is further tuned by an intricate regulatory ensemble that includes phosphatidylinositols (e.g., PIP_2_), calmodulin, protein kinases such as PKC, and scaffold proteins like INAD and Homer (Venkatachal et al., 2007). Through these interactions, TRP channels function as dynamic signaling hubs, integrating diverse physicochemical cues to shape cellular physiology and pathology. The expression profiles of these TRP channels within the cochlea are highly cell-type-specific, forming a complex regulatory network that underlies their diverse functions. A schematic summary of the distribution of key TRP channels in the organ of Corti is presented in Figure 1.

In summary, the structural diversity, polymodal activation, and finely tuned regulation of TRP channels-together with their strategic expression throughout the auditory system-provide a molecular scaffold for their involvement in ototoxic injury. Elucidating the distinct roles of individual TRP isoforms and dissecting their cooperative or antagonistic interactions in cochlear cells will be essential for clarifying their contributions to hearing loss and for designing precise, TRP-targeted therapeutic strategies.

### 3.2 TRP channel-mediated cisplatin ototoxicity

**FIGURE 2:**
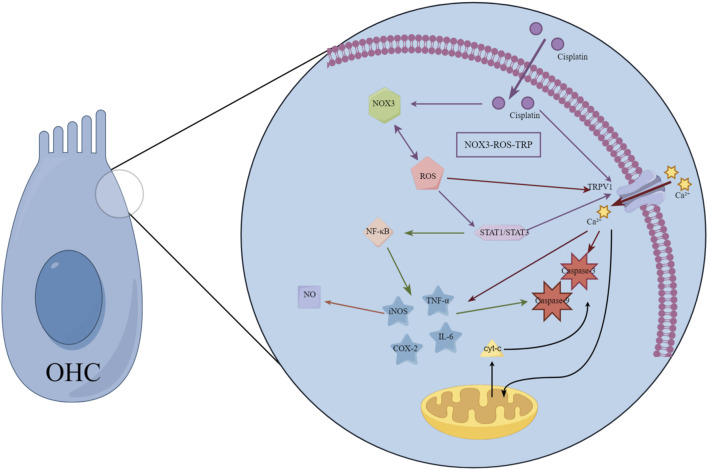
Schematic diagram of cisplatin ototoxicity signaling mediated by TRP channels. This diagram illustrates a positive feedback loop centered on the TRPV1 channel, which amplifies cisplatin-induced hair cell damage. Cisplatin administration increases expression and activates NADPH oxidase 3 (NOX3), triggering a surge of reactive oxygen species (ROS). ROS directly activates TRPV1 channels, triggering calcium influx. The resulting calcium overload exacerbates mitochondrial dysfunction and activates apoptotic pathways. Concurrently, ROS and calcium signals synergistically activate transcription factors STAT1 and NF-κB, driving the production of pro-inflammatory cytokines (e.g., TNF-α, IL-6), which sustain the activation of TRPV1 and NOX3. This self-sustaining “NOX3-ROS-TRPV1” cycle continuously amplifies oxidative stress and inflammatory responses, ultimately leading to hair cell death. This model reveals TRP channels as key drivers and amplifiers of ototoxicity, positioning them as important therapeutic targets.

TRPV1 channel activation serves as a critical facilitator for cisplatin accumulation within cochlear hair cells. After cisplatin enters cells *via* specific transporters such as OCT2, TRPV1 activation triggers a cascade of events–including Ca^2+^ overload and oxidative stress–that potently augment cellular uptake and trap the drug intracellularly, thereby precipitating ototoxicity (Hsieh et al., 2024). Correspondingly, the high basal expression of TRPV1 in outer hair cells (OHCs) parallels their heightened vulnerability; cisplatin inflicts markedly greater damage on OHCs than on the more resilient IHCs (Zheng et al., 2003). Upon entering the cochlea, cisplatin accumulates in hair cells in a dose- and time-dependent manner and unleashes a torrent of oxidative injury. TRPV1 serves not only as a passive conduit for cisplatin but also as a hub in a self-amplifying toxicological circuit. Cisplatin administration increases expression of NOX3 and TRPV1 in the cochlea. These changes, in turn, enhance accumulation of ROS and intracellular Ca^2+^ *via* NOX3 and TRPV1, respectively. The increased ROS further activates and induces TRPV1 and NOX3, contributing to enhanced Ca^2+^ influx into cells. This forms a “NOX3-ROS-TRPV1” positive feedback loop that continuously amplifies hair cell death effects (Mukherjea et al., 2008; Mukherjea et al., 2011). The intricate interplay between these pathways is schematically summarized in Figure 2.

Simultaneously, the ROS generated through this axis also act as potent signaling molecules. Via STAT1, they markedly elevate the cochlear expression of iNOS, COX-2, and TNF-α (Medzhitov and Horng, 2009; Watanabe et al., 2000; Hoshino et al., 2008; Haake et al., 2009), fueling inflammation and transient threshold shifts (Mukherjea et al., 2010). Simultaneously, ROS directly gate TRPV1, provoking Ca^2+^ overload, mitochondrial depolarization, and activation of caspase-3–mediated apoptosis (Mukherjea et al., 2008; Dhukhwa et al., 2019). The ensuing Ca^2+^ surge further releases TNF-α and IL-6, perpetuating an “oxidative stress–inflammation–cell death” vicious cycle. TRPV1 also orchestrates a bidirectional amplification loop between ROS and inflammatory mediators. Activated STAT1 enhances NF-κB signaling, which drives additional pro-inflammatory cytokine release, activates caspases-3 and -9, upregulates iNOS, and boosts nitric oxide production-culminating in cellular scorching and irreversible hearing loss (Hazlitt et al., 2018). Direct evidence for TRPV1’s centrality comes from intratympanic delivery of TRPV1 siRNA, which reduces cochlear TRPV1 protein by ∼85% and markedly attenuates cisplatin-induced auditory brainstem response (ABR) threshold elevations (Mukherjea et al., 2008). Emerging evidence indicates that TRPV4 may cooperate with TRPV1 to facilitate cisplatin entry, although the precise molecular interactions remain to be elucidated (Ramkumar et al., 2022). Importantly, the gating of TRP channels is reciprocally modulated by inflammatory and oxidative milieus. Within this framework, cisplatin-activated STAT1 amplifies TRPV1-driven inflammation, whereas STAT3 signaling-potentially *via* CB2 receptor engagement-exerts a counterbalancing, cytoprotective effect (Bhatta et al., 2019). These mechanistic insights consolidate the rationale for precision otoprotection strategies that selectively modulate TRP channel activity and their downstream transcriptional partners.

### 3.3 Regulatory role of TRP channels in cochlear hair cells

TRP channels form an intricate and finely tuned regulatory network within the cochlea. By detecting diverse physicochemical cues and modulating Ca^2+^ dynamics, they orchestrate mechano-electrical transduction, preserve cellular homeostasis, guide developmental maturation, and coordinate adaptive or maladaptive responses to noise, aging, and ototoxic drugs. Their actions are cell-type-specific, yet individual isoforms can compensate for or synergize with one another, conferring context-dependent roles that range from cytoprotection to injury amplification. Precise mapping of each TRP channel’s function in discrete cochlear cell types-and clarification of their inter-subtype interactions-will provide the mechanistic bedrock for next-generation, precision hearing-protection therapies.

TRPV subunits display a remarkably cell-specific topography throughout the cochlea. TRPV1, -3, and -4 transcripts are abundant in SGNs, hair cells, and SV (Ishibashi et al., 2008; Yamauchi et al., 2010; Wang et al., 2019). TRPV3 is conspicuously restricted to the cytosol of OHCs, with little or no localization to the stereociliary bundle (Ishibashi et al., 2008). TRPV4, in addition to labeling hair cells, is strongly expressed in SV marginal cells and in supporting cells of the organ of Corti, implicating it in the regulation of endolymphatic ion balance and osmotic equilibrium (Shen et al., 2006; Tabuchi et al., 2005). TRPC channels-TRPC1, -3, -4, -5, -6, and -7-are broadly distributed in SGNs, SV, and vestibular end organs (Takumida and Anniko, 2009). TRPC3 is particularly notable: it is highly expressed in hair cells during embryonic and early postnatal life, then markedly upregulated in SGNs from the third postnatal week onward, implying a critical role in cochlear maturation (Phan et al., 2010). TRPC6 has also been localized to the organ of Corti and SGNs in the human cochlea (Eng et al., 2023). TRPM4 exhibits a spatiotemporally precise expression pattern in the murine cochlea: it is markedly upregulated on postnatal day 14-the developmental stage when auditory function first emerges-and is restricted to the apical border cells of the SV, the cytoplasm of IHCs, and a subset of type II SGNs. This distribution implicates TRPM4 in both the maturation of the auditory system and the maintenance of the inner ear’s ionic and osmotic homeostasis (Sakuraba et al., 2014). TRPML3 resides in the endolysosomal compartments of hair cells, strial marginal cells, and vestibular endothelial cells, as well as at the base of stereocilia, where it governs lysosomal trafficking and organellar homeostasis (Casti et al., 2011). TRPA1 is concentrated in non-sensory supporting cells-Hensen, Deiters, and pillar cells-rather than in hair cells themselves (Corey et al., 2004; Vélez-Ortega et al., 2023).

While the spatiotemporal expression patterns of these TRP channels are well-delineated,their specific roles in the pathological context of cisplatin ototoxicity warrant deeper critical evaluation. Beyond the central role of TRPV1, other TRP family members are poised to act as important modifiers of ototoxic damage through their unique activation mechanisms. This is consistent with their well-established roles as bidirectional stress sensors in the cochlea, capable of pivoting toward either protection or toxicity depending on the context. For instance: TRPA1, a renowned sensor of oxidative stress and inflammatory mediators (Kim and Hwang, 2013), is densely expressed in non-sensory supporting cells. Cisplatin-induced ROS and cytokine release could therefore activate TRPA1, triggering Ca^2+^ influx that alters the contractile state of Deiters' and pillar cells, thereby modulating cochlear micromechanics and potentially influencing hair cell vulnerability [This speculative role aligns with its known activation by noise and ototoxic drugs (Vélez-Ortega et al., 2023)]. Similarly, the endolysosomal TRPML3 channel, crucial for organellar homeostasis (Miao et al., 2015), may see its function compromised by cisplatin-induced stress, impairing autophagic flux and exacerbating injury.

This Janus-faced nature of TRP channels is exemplified by specific findings: Compensatory TRPV4 upregulation in OHCs of TRPV3-knockout mice confers resistance to ototoxic drugs (Wang et al., 2019), demonstrating how one channel’s loss can be mitigated by another. Noise upregulates TRPV1 and synergizes with TNF-α to amplify Ca^2+^ influx, inflammation, and permanent threshold elevation (Dhukhwa et al., 2019), illustrating a maladaptive, pro-inflammatory response. Conversely, the TRPML3-A419P gain-of-function mutation (varitint-waddler mice) causes constitutive channel opening, Ca^2+^ overload, and neonatal hair-cell death (Grimm et al., 2007), while combined TRPML1/3 knockdown triggers early-onset progressive hearing loss with lysosomal failure (Wiwatpanit et al., 2018), revealing their critical redundant roles in maintaining cellular integrity.

Collectively, TRP channels constitute a versatile Ca^2+^-signaling network that maintains cochlear homeostasis under physiological conditions yet can pivot toward either protection or toxicity under stress. Precise delineation of their cell-specific functions and inter-channel crosstalk is essential for designing targeted therapies that exploit their protective potential while minimizing their pathogenic activation. Capitalizing on the pivotal roles of TRP channels in cochlear physiology and pathology, these proteins have become prime therapeutic targets for hearing preservation. Fine-tuning TRPA1 activity in supporting cells offers a promising avenue for accelerating auditory recovery after noise trauma (Vélez-Ortega et al., 2023). Meanwhile, pharmacological enhancement of TRPML-mediated lysosomal clearance may slow the inexorable progression of age-related hearing loss by sustaining proteostasis and cellular resilience (Wiwatpanit et al., 2018). Preclinical studies indicate that drugs protecting cochlear hair cells from damage by TRP channels include natural product extracts targeting TRPV1 (such as Puerarin and Piparine), traditional Chinese medicine compounds (TS), synthetic small molecules (such as Ursolic acid), and Clonidine targeting TRPC6 (Lin et al., 2024; Zallocchi et al., 2024; Di et al., 2020; Hong et al., 2023; Zhai et al., 2025). A comprehensive summary of these TRP-channel-targeted pharmacological agents, their mechanisms of action, and corresponding references is provided in Table 1.

**TABLE 1:** TRP channel-targeted drugs to protect cochlear hair cell damage.

Drugs	Targeting TRP channels	Mechanism	References
Ursolic acid	TRPV1	Prevention of cisplatin-induced ototoxicity by inhibiting oxidative stress and TRPV1-mediated Ca^2+^ signaling in a mouse model	Di et al. (2020)
A combination of Cuscutae Semen and Rehmanniae Radix Preparata (TS)	TRPV1	A combination of Cuscutae Semen seeds and Rehmanniae Radix Preparata (TS) ameliorates sensorineural hearing loss by modulating the expression of Trpv1, Cacna1h, and Ngf genes in zebrafish and mouse models.	Hong et al. (2023)
Piplartine	TRPV1	Prevents aminoglycoside ototoxicity in mice, possibly by modulating TRPV1 expression and directly or indirectly modulating AKT1 activity in a mouse model	Zallocchi et al. (2024)
Puerarin	TRPV1	Inhibition of the TRPV1/IPR3/p65 pathway blocks calcium overload and excessive ROS expression in a mouse model, ameliorating cisplatin-induced ototoxicity and apoptosis	Lin et al. (2024)
Clonidine	TRPC6	Inhibits norepinephrine release and blocks TRPC6 channel-mediated Ca^2+^ inward flow in cochlear hair cells, which in turn inhibits the MLCK-MRLC signaling pathway and significantly attenuates hair cell damage and synaptic degeneration caused by noise exposure in a mouse model.	Zhai et al. (2025)

## 4 Clinical dilemmas and future directions

The strategic targeting of TRP channels, while promising, is not without its own set of unique challenges that must be squarely addressed. Foremost among these is the issue of on-target, off-organ toxicity. TRP channels are ubiquitously expressed and mediate critical physiological processes throughout the body; systemic inhibition of TRPV1, for example, could impair thermoregulation and pain perception (Tóth et al., 2011), while affecting TRPM8 may disrupt cold sensing (Dhaka et al., 2007). Furthermore, functional redundancy within the TRP family means that blocking one channel might be compensated by another, potentially diminishing therapeutic efficacy. The current lack of highly selective agonists/antagonists for specific TRP subtypes further complicates the landscape, raising the risk of off-target effects. These inherent challenges underscore the fact that simply identifying a target is insufficient-innovative delivery and targeting strategies are paramount to success. It is within this context of target-specific challenges and the broader limitations of existing clinical approaches that the path forward must be charted. Despite remarkable advances in deciphering cisplatin-induced ototoxicity, translation into safe and effective clinical therapies remains fraught with obstacles. Current protective paradigms-centered on antioxidants, anti-inflammatory agents, and local drug delivery-each carry substantial limitations. Systemic antioxidants such as N-acetylcysteine or D-methionine indiscriminately scavenge ROS, risking attenuation of cisplatin’s antitumor activity, while the BLB further restricts their cochlear bioavailability (Shen et al., 2023). Anti-inflammatory strategies (e.g., STAT1 inhibition with EGCG or TNF-α blockade *via* etanercept) mitigate cochlear injury in pre-clinical models, yet systemic immunosuppression raises concerns for prolonged clinical use (Dhukhwa et al., 2019; Borse et al., 2017). Intratympanic administration bypasses systemic exposure, but the round-window membrane’s low permeability, procedural invasiveness, and uneven drug distribution across the cochlear turns severely curtail efficacy and patient acceptance (Mukherjea et al., 2008). Consequently, available interventions exhibit wide inter-individual variability, lack long-term safety data, and, as exemplified by the cytoprotectant amifostine, can yield unacceptable neurotoxicity in pediatric populations (Church et al., 2004).

Targeting TRP channels-key gatekeepers of cisplatin entry and amplifiers of cochlear injury-offers a compelling next-generation otoprotective paradigm. Structure-guided design of small-molecule inhibitors or allosteric antagonists directed at the pore-forming loop or regulatory domain of TRPV1 can block cisplatin influx with high precision while sparing other physiological channel functions and minimizing off-target effects. Encapsulating these agents-or complementary protectants such as antioxidants, anti-inflammatories, or calcium-channel blockers-in engineered nanoparticles (liposomes, polymeric carriers) equipped with cochlear-homing ligands promises to bypass the blood–labyrinth and round-window barriers, concentrating therapy within the inner ear and limiting systemic exposure and interference with cisplatin’s antitumor activity (Xie et al., 2023; Chen et al., 2019). Because cisplatin ototoxicity arises from convergent oxidative, inflammatory, and mitochondrial pathways, monotherapy is unlikely to suffice. Future regimens should integrate TRP-channel blockade with complementary strategies-e.g., autophagy enhancement, pyroptosis inhibition, and mitochondrial protection-to harness synergistic multi-pathway defense. Robust translation will require close coupling of basic science with clinical medicine: refinement of predictive pre-clinical models, rigorous multicenter trials, and prioritized evaluation in pediatric patients at highest risk. The ultimate goal is to establish personalized, chemo-fidelity-sparing protocols that safeguard hearing without compromising oncologic efficacy.

In summary, leveraging TRP channels as the linchpin and coupling them with state-of-the-art targeted-delivery technologies offers a realistic path to resolve the current clinical impasse in preventing cisplatin ototoxicity-achieving the dual imperative of maximizing chemotherapeutic efficacy while safeguarding patients’ hearing.
